# Attenuated Oxidative Stress following Acute Exhaustive Swimming Exercise Was Accompanied with Modified Gene Expression Profiles of Apoptosis in the Skeletal Muscle of Mice

**DOI:** 10.1155/2016/8381242

**Published:** 2016-04-10

**Authors:** Yi Sun, Di Cui, Zhe Zhang, Tan Zhang, Jun Shi, Haixiu Jin, Zhe Ge, Liu Ji, Shuzhe Ding

**Affiliations:** ^1^Key Laboratory of Adolescent Health Assessment and Exercise Intervention of Ministry of Education, East China Normal University, Shanghai 200241, China; ^2^School of Physical Education & Health Care, East China Normal University, Shanghai 200241, China

## Abstract

*Purpose*. The purpose of the present study was to investigate the effect of acute exhaustive swimming exercise on apoptosis in the skeletal muscle of mice.* Method*. C57BL/6 mice were averagely divided into seven groups. One group was used as control (C), while the remaining six groups went through one-time exhaustive swimming exercise and were terminated at 0 h, 2 h, 6 h, 12 h, 24 h, and 48 h upon completion of exercise.* Result*. ABTS was significantly lowered at 12 h and 48 h after exercise. The MDA level was significantly decreased at any time points sampled following exercise. Total SOD activity was significantly decreased at 6 h, 12 h, 24 h, and 48 h after exercise. Neither mRNA of Bax nor Bax/Bcl-2 ratio was significantly altered by exercise. mRNA of Bcl-2 was significantly decreased since 6 h after exercise. mRNA and protein expressions of PGC-1*α* were significantly increased at different time points following exercise.* Conclusion*. Cellular oxidative stress level was decreased following low intensity, long duration acute exhaustive swimming exercise in mice, and the enzymatic antioxidant capacity was compromised. Apoptosis of the skeletal muscle was inhibited, which could partially be explained by the enhanced level of PGC-1*α*.

## 1. Introduction

Free radical is species that contains one or more unpaired electrons in the systems, which is mostly derived from oxygen or nitrogen [1]. Oxidative stress is a condition that the production and clearance of free radicals are unbalanced and that the clearance rate of free radicals is not sufficient to meet their production [2]. Excessive production of reactive oxygen and nitrogen species (RONS) could happen under various conditions and by different stimulants, such as toxins and pollutants and excessive intake of nutrients and exercise. In order to keep the delicate balance between production and clearance of RONS, the body's antioxidant defense system needs to be activated to protect cells, therefore playing a vital key role in regulating cellular functions. Even though oxidative stress is usually needed to initiate signaling pathways, a shift back to the reducing condition is eventually required. When the antioxidant defense system is not strong and efficient enough to counterbalance the accelerated production of RONS, a long-lasting condition of oxidative stress could be caused, thus disturbing cellular signaling pathways. A permanent shift in the redox balance could also bring damage to cellular substrates such as nucleic acids, lipids, and proteins and eventually cause cell death via apoptosis.

As a condition that could trigger the generation of RONS, the effect of exercise on oxidative stress level depends on energy requirements, oxygen consumption, and physiological stress imposed. No wonder that exercise of different frequency, intensity, duration, and type could result in various production rates of RONS. The general idea is that during exercise of low intensity and duration, the body's antioxidant defense system is usually strong enough to clear RONS production. However, following intense or prolonged exercise, oxidative damage is usually seen because of the compromised antioxidant defense system. However, when it comes to the effect of acute exhaustive exercise on oxidative stress, discrepancy still exists in the findings. For example, after performing an acute period of exhaustive exercise on cycle ergometer [3], following either half-Ironman triathlon, full-Ironman triathlon [4], or cycling mountain stage of 171.8 km [5], a significant increase in MDA was observed in blood. However, some other studies reported no significant change in the biomarkers of oxidative stress following acute exercise, for example, after maximal cycling to fatigue [6], 21 km run [7], or exercising the knee isokinetically to exhaustion [8]. In addition, even very rarely, decreased MDA level following acute exhaustive exercise has been reported in rats [9]. Similar finding has also been observed in human following acute submaximal exercise [10]. Based on the above previous studies, the authors wonder whether the nature of very low intensity and long duration of acute exhaustive swimming exercise could be the reason of some unusual observations of decreased MDA level following exhaustive exercise, as reported by Balci et al. [9], and whether similar finding could be observed in other animal models such as mice.

Apoptosis is a highly and tightly regulated process that ensures the tissue and cellular homeostasis of multicellular organisms. The signaling pathways of apoptosis in mammalians are sensitive to the intracellular redox environment and thus can be either activated or deactivated by oxidative stress. Therefore, the cellular signaling pathways of apoptosis could be directly or indirectly influenced by exercise via disturbance of the redox balance [11]. Regular exercise has been demonstrated to alleviate apoptosis and DNA fragmentation in the skeletal muscle, whose effects could be mediated by several potential mechanisms, including altering the gene and protein expression of several apoptotic proteins such as Bax, Bcl-2, and caspases. On the contrary, acute exercise is generally accepted to be associated with elevated DNA fragmentation and augmented apoptosis [12]. However, it is to be explored how apoptosis could be affected and regulated by acute exhaustive swimming exercise in animal model settings, in which condition oxidative stress level was possibly alleviated. Besides, as a key regulator of mitochondrial biogenesis, peroxisome proliferator-activated receptor *γ* coactivator-1*α* (PGC-1*α*) is considered to be affected by different modes of physical activity. Both acute exercise and chronic training have been demonstrated to upregulate the expression of PGC-1*α* [13, 14]. Since mitochondrial pathway is known to be the main mediator of apoptosis, the potential role of PGC-1*α* in apoptosis following acute exhaustive swimming exercise is also to be examined.

Therefore, the purpose of the present study was to investigate the effect of acute exhaustive swimming exercise on apoptosis in the skeletal muscle of mice as well as explore the possible role of oxidative stress, antioxidant capacity, and PGC-1*α* in this process.

## 2. Materials and Methods

All experimental protocols employed were approved by the Committee on the Care of Laboratory Animal Resources, East China Normal University, and were conducted in accordance with the Guide for the Care and Use of Laboratory Animals.

### 2.1. Animals

Forty-two clean grade male C57BL/6 mice (16.9 g–22.5 g, Shanghai SLAC Laboratory Animal Co., Ltd., China) were used. They were housed in cages maintained at 22 ± 1°C and 50 ± 5% humidity with 12 h light/dark cycle. Standard chow and water were available* ad libitum* before exercise.

### 2.2. Acute Exhaustive Swimming Exercise

A forced swimming pool especially planned for mice was designed. The tank is a glass chamber filled with water 50 cm high. The temperature of the water within the tank was kept at 30 ± 2°C via a thermostatically controlled heater located by the side of the tank. During the first week, all the mice were accustomed to swimming with repeated short-term swimming sessions for five days. After two-day break, the mice were randomly divided into seven groups, with six in each group. One group was used as control (C), and mice of the remaining six groups were put through one-time exhaustive swimming exercise. Exercise was terminated when the mice stayed submerged for 10 s. Mice of Group C were euthanized without exercise intervention, while mice of the exercise groups were euthanized 0 h, 2 h, 6 h, 12 h, 24 h, and 48 h after completion of the swimming exercise, respectively, and were called Group 0, Group 2, Group 6, Group 12, Group 24, and Group 48, correspondingly.

### 2.3. Tissue Harvesting

Mice of the intervention groups were terminated by cervical dislocation 0 h, 2 h, 6 h, 12 h, 24 h, and 48 h upon completion of exhaustive exercise, respectively, while mice of Group C were terminated with the same method after fasting for 24 hours. The gastrocnemius (GS) muscles of both hindlimbs were carefully isolated, rinsed with saline, and weighed. The tissue was snap frozen in liquid nitrogen and further stored at −80°C.

### 2.4. Assays of Oxidative Stress and Antioxidant Capacity

The GS was weighed and homogenized in PBS of 4°C, followed by centrifugation at 12000 g for 5 min to get the supernatant for further test. Malondialdehyde (MDA) content, superoxide dismutase activity (Mn-SOD, Cu-Zn SOD, and t-SOD), and catalase (CAT) activity were determined with commercial assay kits produced by Nanjing Jiancheng Bioengineering Institute, China. Total antioxidant capacity was measured by ABTS assay kits purchased from Beyotime Biotechnology, China. The total protein content of each sample was measured by the Bradford method to normalize the results.

### 2.5. RNA Extraction and Quantitative Real-Time PCR (qRT-PCR)

About 20 mg of tissue samples was homogenized for extraction of total RNA and DNA with TRizol assay kit. Specific primers for Bax, Bcl-2, Caspase 3, Bcl-XL, and PGC-1*α* were designed with primer 3. The primer sequences were as follows: Bax: forward 5′-CGA ATT GGA GAT GAA CTG GAC AG-3′ and reverse 5′-CTA GCA AAG TAG AAG AGG GCA AC-3′; Bcl-2: forward 5′-TTG TAA TTC ATC TGC CGC CG-3′ and reverse 5′-AAT GAA TCG GGA GTT GGG GT-3′; Bcl-XL: forward 5′-GAC CGC GTA TCA GAG CTT TG-3′ and reverse 5′-GCT GCA TTG TTC CCG TAG AG-3′; and PGC-1*α*: forward 5′-AAG TGT GGA ACT CTC TGG AAC TG-3′ and reverse 5′-GGG TTA TCT TGG TTG GCT TTA TG-3′. RNA was quantified by spectrophotometry (260 nm). cDNA was prepared by reverse transcription of 5 *μ*L total RNA using ReverTra Ace qPCR-RT Kit (Toyobo, FSQ-101). qRT-PCR was continued on StepOne instrument (ABI) and software (Applied Biosystems) using SYBR Green PCR Master Mix (Toyobo, QPK-201) for detection. The thermocycling conditions for PCR were as follows: 95°C for 1 min, followed by 40 cycles of 95°C for 15 s, 61°C for 30 s, and 72°C for 45 s. All samples were run in duplicate and data were analyzed according to 2^−ΔΔCT^ method with GAPDH as an internal control. The identity and purity of the amplified product were checked through analysis of the melting curve carried out at the end of amplification. A value of 1 was arbitrarily assigned to Group C to which values of Groups 0, 2, 6, 12, 24, and 48 were reported.

### 2.6. Western Blotting

The GS was homogenized in lysis buffer containing 50 mM Tris HCl, 150 mM NaCl, 1 mM EDTA, 0.2 mM PMSF, and 1% NP-40, with pH of 7.4. The homogenate was centrifuged at 8000 g for 10 min at 4°C. The protein concentration of the supernatant was determined with the method of BCA. 10% SDS-PAGE was used to separate protein, which was then blotted on PVDF membrane. The blots were incubated with primary antibodies (1 : 200, Santa Cruz) overnight at 4°C. The membranes were incubated with secondary antibodies (1 : 500, Santa Cruz) for 1 h at room temperature. The membrane was examined with ECL Plus (high sensitivity ECL chemiluminescence kit). GAPDH was used as a control to ensure equal protein loading on the gel, and the blots were quantified with Alphatech Gel Imager.

### 2.7. Caspase 3 Activity

Caspase 3 activity in the skeletal muscle was measured using Caspase 3 Activity Assay Kit, according to the manufacturer's instruction (Beyotime Biotechnology, China). Briefly, after homogenizing the muscle sample in cell lysis, the homogenates were centrifuged for 10 min at 15000 g; the supernatant was then incubated with Ac-DEVD-pNA and reaction buffer for 90 min at 37°C. Absorbance was measured at 405 nm. An increase in OD_405_ indicated activation of Caspase 3.

### 2.8. Statistical Analysis

Results were expressed as mean ± SE. The difference between groups was tested for significance with one-way ANOVA using GraphPad Prism 6. The significance threshold was set to *p* < 0.05.

## 3. Results

### 3.1. Body Weight and Exhaustion Time

**Figure 1:**
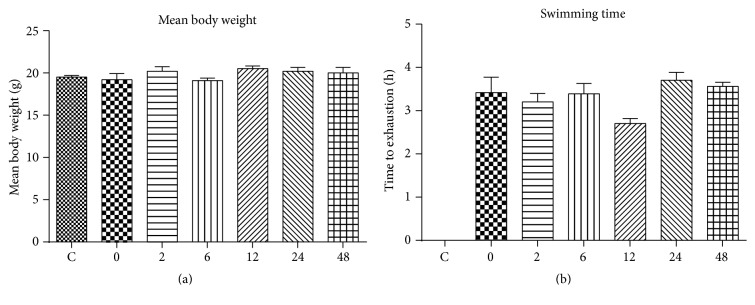
The effect of acute exhaustive swimming exercise on the body weight (a) and swimming time (b) of the mice.

The body weight and time to exhaustion of the mice are displayed in Figures 1(a) and 1(b), respectively. The body weight of Group 0 through Group 48 was not significantly different from Group C. Likewise, the exhaustion times of Group 0 through Group 48 were not significantly different from each other.

### 3.2. Oxidative Stress

**Figure 2:**
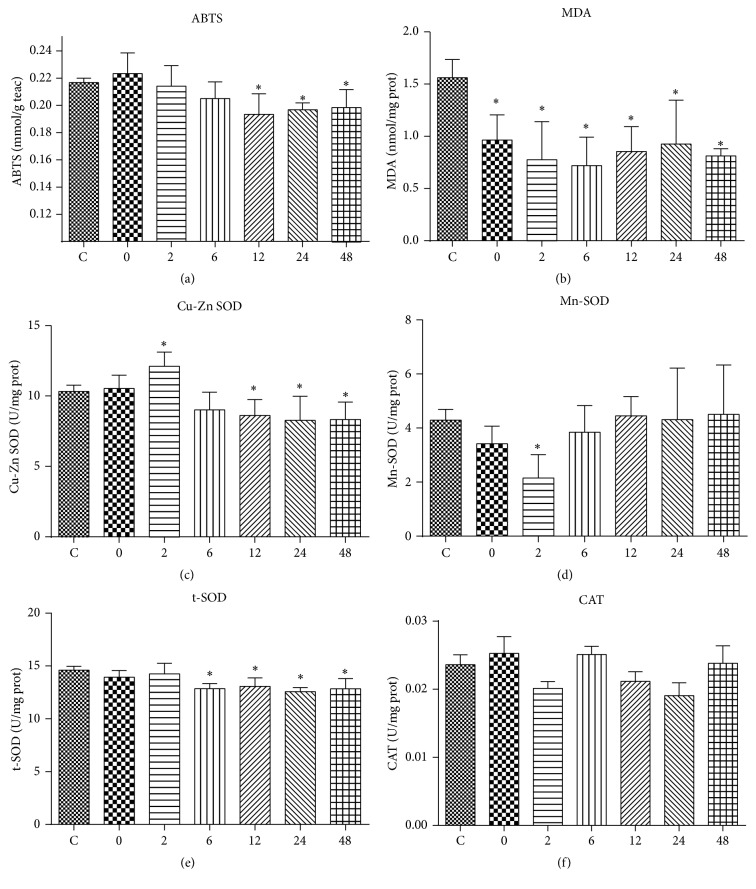
The effect of acute exhaustive swimming exercise on ABTS (a), MDA content (b), Cu-Zn SOD activity (c), Mn-SOD activity (d), t-SOD activity (e), and CAT activity (f). Significant difference between Group C and other groups: ^*∗*^ *p* < 0.05.

The result of ABTS is displayed in Figure 2(a). ABTS of Group 12 through Group 48 was significantly lower compared to Group C. The MDA level (Figure 2(b)) of Group 0 through Group 48 (0.71–0.96 mmol/mg prot) was significantly lower compared to Group C (1.56 mmol/mg prot). Cu-Zn SOD (Figure 2(c)) of Group 2 was significantly higher compared to Group C, while Cu-Zn SOD of Groups 12, 24, and 48 was significantly lower compared to Group C. As for Mn-SOD (Figure 2(d)), the value of Group 2 (2.15 U/mg prot) was significantly lower compared to Group C (4.29 U/mg prot). Total SOD (Figure 2(e)) of Groups 6, 12, 24, and 48 was significantly lower compared to Group C. Catalase activities of the seven groups were not significantly different from each other (Figure 2(f)).

### 3.3. qRT-PCR

**Figure 3:**
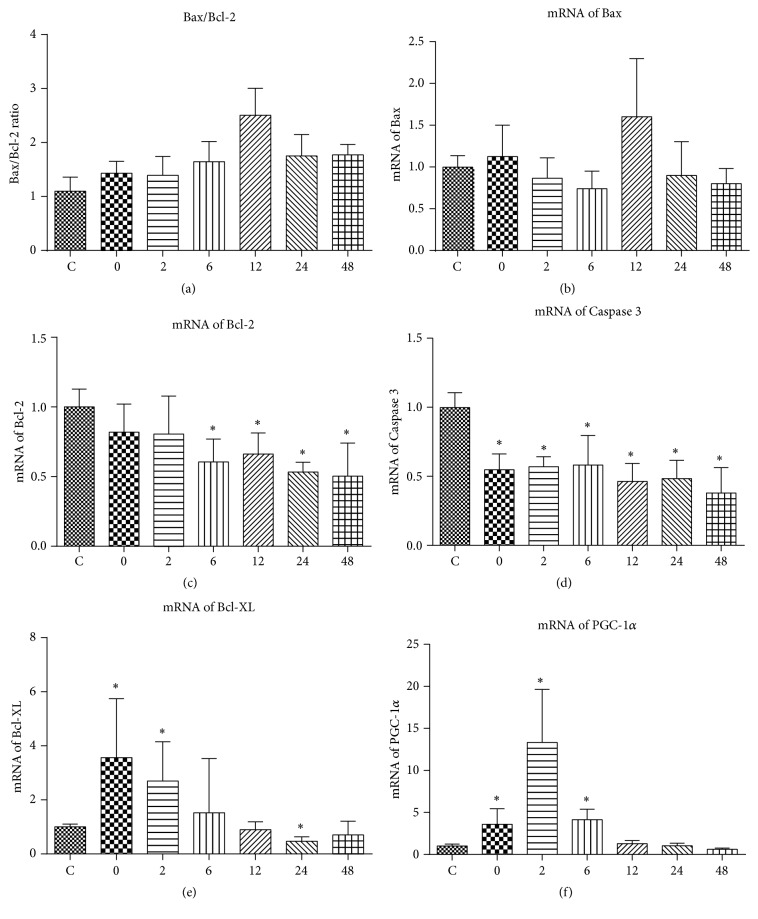
The effect of acute exhaustive swimming exercise on the mRNA expressions of Bax/Bcl-2 (a), Bax (b), Bcl-2 (c), Caspase 3 (d), Bcl-XL (e), and PGC-1*α* (f). Significant difference between Group C and other groups: ^*∗*^ *p* < 0.05.

Neither mRNA of Bax (Figure 3(a)) nor Bax/Bcl-2 ratio (Figure 3(b)) was significantly altered by acute exhaustive swimming exercise. However, mRNA of Bcl-2 in Group 6 through Group 48 was significantly decreased compared to Group C (Figure 3(c)). mRNA of Caspase 3 was demonstrated to be decreased at any time points collected following exercise (Figure 3(d)). mRNA of Bcl-XL was significantly increased in Groups 0 and 2 while it was significantly decreased in Group 24 compared to Group C (Figure 3(e)). mRNA of PGC-1*α* was significantly increased in Group 0 through Group 6 compared to the control group (Figure 3(f)).

### 3.4. Caspase 3 Activity

**Figure 4:**
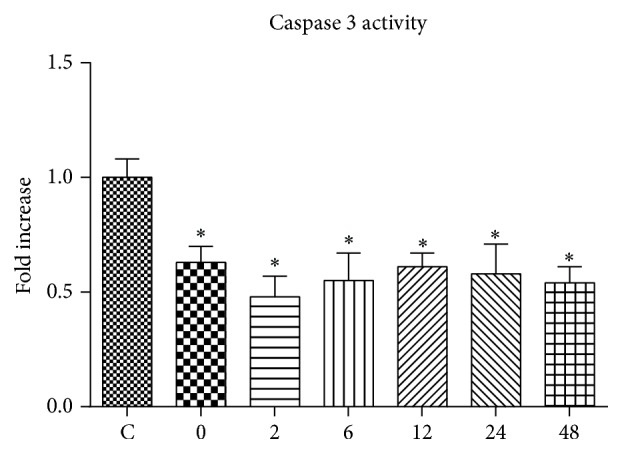
The effect of acute exhaustive swimming exercise on Caspase 3 activity. Significant difference between Group C and other groups: ^*∗*^ *p* < 0.05.

The Caspase 3 activity was significantly decreased at 0 h, 2 h, 6 h, 12 h, 24 h, and 48 h after exercise (Figure 4).

### 3.5. Western Blot

**Figure 5:**
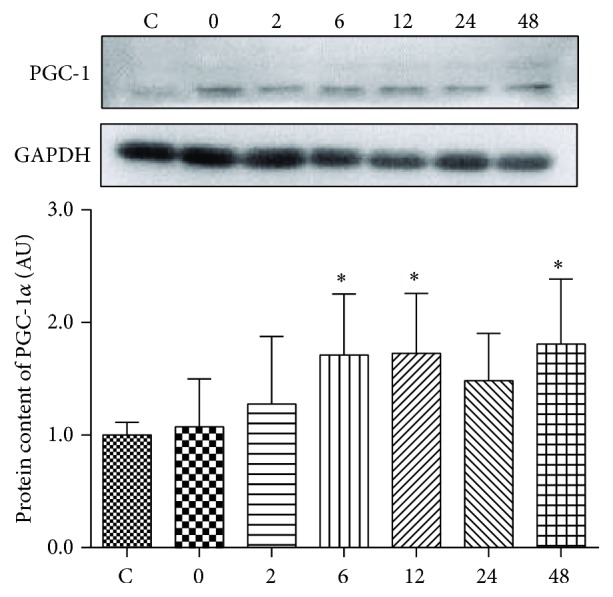
The effect of acute exhaustive swimming exercise on the protein content of PGC-1*α*, normalized by GAPDH. Significant difference between Group C and other groups: ^*∗*^ *p* < 0.05.

The protein content of PGC-1*α* was significantly increased at 6 h (1.7-fold), 12 h (1.7-fold), and 48 h (1.8-fold) after exercise (Figure 5).

## 4. Discussion

The main finding of the present study was that the intensity and duration of exercise were key to determining the effect of acute exhaustive exercise on oxidative stress and apoptosis. Even though numerous studies reported that strenuous exercise could cause an increase in the oxidation of lipid, protein, and DNA, the present study suggested that the MDA level of the acute exhaustive swimming exercise group was lower compared to the control group. In agreement with the present findings, Balci et al. also reported that the MDA level in the exhausted groups was lower compared to the remaining group in trained rats [9]. Therefore, even though rarely reported, it could be postulated that the decreased MDA level (or even cellular oxidative stress) was due to the low intensity and long duration nature of exhaustive swimming exercise implemented, compared to exhaustive treadmill exercise. Swimming was selected in the present study because it is less traumatic to the animals. Electrical shock is usually used as a stimulator to force the mice to run in treadmill-based exercise protocols. However, the electrical shock itself could be the trigger of oxidative stress. In contrast to the present study, other investigations have yielded different outcome with regard to MDA level following exhaustive exercise. For example, Gul et al. [15] and Liu et al. [16] reported that the MDA levels of heart and skeletal muscle were not affected by acute exhaustive treadmill exercise. Others have observed an increase in MDA level of the heart tissue following exhaustive swimming exercise in rats [17, 18].

When it comes to antioxidant capacity in response to exercise intervention, the results of investigations vary too. The general idea is that acute exercise usually results in a great increase in enzyme activities, possibly to compensate the elevated production of superoxides and oxyradicals [19]. However, CAT activity was sometimes reported as not changed [3] or even decreased [4, 20] after performing acute exercise. In the present study, CAT activity was not significantly changed at any time points collected following exhaustive swimming exercise. This suggests that the fluctuation of CAT activity does not necessarily follow the same pattern of alteration of MDA level. However, as a measurement reflecting the total antioxidant capacity of tissues, ABTS was significantly decreased 12 h, 24 h, and 48 h after exercise. It is needed to point out that the finding of the present study does not necessarily suggest that the antioxidant capacity of mice was improved after exercise. We could not rule out the possibility that the acute, long-lasting, and low intensity exercise could induce an early adaptive response in GS, which stabilized thereafter. In order to verify this, further studies with the same animal model and samples collected at more time points from the start of exercise should be carried out.

Major antioxidant enzymes such as SOD are regarded as the first line of the antioxidant defense system against ROS during oxidative stress. However, there have been many conflicting studies concerning the change of SOD in response to acute exercise. As an endogenous antioxidant enzyme, SOD activity has been reported to be either increased [21], decreased [22], or not changed [23] following acute exhaustive treadmill exercise in rats. In the present study, the activity of total SOD was significantly decreased 6 h through 48 h after exercise. The decreased SOD activity is in line with the decreased MDA level, suggesting that the antioxidant capacity of the mice could be attenuated after exercise, possibly due to the low intensity nature of unloaded swimming exercise. Of the three isoforms of SOD that could catalyze the dismutation of superoxide anions to H_2_O_2_, Cu-Zn SOD is located mainly in the cytosol and nucleus and to a lesser extent in the mitochondria [24]. In contrast, Mn-SOD is primarily located in the mitochondrial matrix and therefore is the main isoform that catalyzes the dismutation of superoxide anions generated by the respiratory chain [25]. The changes of Cu-Zn SOD and Mn-SOD are not always paralleled following exercise intervention. For example, the activity of both Cu-Zn SOD and Mn-SOD in the skeletal muscle was either not changed [26] or increased after a single bout of exercise [27]. However, in another study, Mn-SOD protein content was found increased following treadmill training, while protein content of Cu-Zn SOD was not significantly altered [28]. The different pattern of change of Cu-Zn SOD and Mn-SOD was likely due to their different location. In the present study, Cu-Zn SOD was significantly increased 2 h after exercise and was significantly decreased 12 h, 24 h, and 48 h after exercise while Mn-SOD was only found decreased 2 h after exercise.

The complicated signaling pathways of apoptosis in mammalians are sensitive to and regulated by intracellular redox environment [29]. The mitochondrial pathway is generally considered as the main mediator of apoptosis. Apoptotic pathways in the skeletal muscle could be affected by exercise. Acute exercise in animals has been reported to be associated with enhanced apoptotic level and elevated DNA fragmentation. For example, eccentric type exercise and exhaustive exercise were reported to significantly increase the Bax/Bcl-2 ratio [12, 30, 31]. Both eccentric exercise and acute strenuous treadmill running elevated Caspase 3 in the skeletal muscle [32, 33]. In the present study, the mRNA expression of Bax was not significantly altered by acute exhaustive swimming exercise, neither was the ratio of Bax/Bcl-2. However, the mRNA expression of Bcl-2 was significantly decreased since 6 h after exercise. Besides, the mRNA expression of Bcl-XL was significantly increased at 0 h and 2 h after exercise. Put together, these results suggest that the low intensity and long duration acute exhaustive swimming exercise might have shifted the mRNA expression of apoptotic related proteins towards the more antiapoptotic direction. Indeed, both the mRNA expression and activity of Caspase 3 were significantly decreased by exercise intervention. But, again, we still could not rule out the possibility that acute swimming exercise could have induced an early adaptation in the gene expression profiles of apoptosis, which lasted till completion of the exhaustive exercise.

As mentioned above, the mitochondrial pathway is the key regulator of apoptosis. Therefore, any alteration in mitochondrial content or function could have an impact on the death of muscle fibers. PGC-1*α* is associated with mitochondrial biogenesis and thus could increase mitochondrial content and promote oxidative phosphorylation [34]. Muscle-specific overexpression of PGC-1*α* was demonstrated to alleviate increase in Caspase 3 and DNA fragmentation. Similarly, PGC-1*α* deficient mice had greater release of cytochrome C from mitochondria [35]. In the present study, the mRNA expression of PGC-1*α* was significantly increased at 0 h, 2 h, and 6 h following exercise, which could partially explain the decreased mRNA expression and activity of Caspase 3.

In conclusion, cellular oxidative stress level was decreased following low intensity, long duration acute exhaustive swimming exercise in mice, and the enzymatic antioxidant capacity reflected by activity of total SOD and Cu-Zn SOD was compromised. In accordance with decreased oxidative stress, apoptosis of the skeletal muscle was inhibited, which could partially be explained by the enhanced level of PGC-1*α*.
